# Assessing Different Fruit Formulations for the Supplementation of Bakery Products with Bioactive Micro-Constituents from Sweet Cherry (*Prunus avium* L.) and Sour Cherry (*Prunus cerasus* L.): A Physicochemical and Rheological Approach

**DOI:** 10.3390/foods13172794

**Published:** 2024-09-02

**Authors:** Evangelia D. Karvela, Evgenia N. Nikolaou, Dimitra Tagkouli, Antonia Chiou, Vaios T. Karathanos

**Affiliations:** Department of Nutrition and Dietetics, Harokopion University of Athens, 70, El. Venizelou Ave., 17671 Athens, Greece; ekarvela@hua.gr (E.D.K.); evgenia@hua.gr (E.N.N.); dtagkoul@hua.gr (D.T.); chiou@hua.gr (A.C.)

**Keywords:** enrichment, bakery products, fruit extract, sour cherry, cherry

## Abstract

Sour and sweet cherries were evaluated as functional components in bread-making because of their bioactive microconstituent content. Five forms of enrichment for each fruit, including the hydroalcoholic extract, lyophilized pulverized fruit, lyophilized extract, and their combinations, were used for supplementation. The physicochemical (pH, color, moisture, rheology, and texture) and sensory properties of dough and bread were assessed in different environments (biological and chemical leavening). Sour cherry in pulverized and extract forms showed higher phenolic content than sweet cherry, especially in the pulverized form. The viscoelasticity of the doughs varied based on the proofing environment and the fortification form. Chemically leavened doughs exhibited higher moduli (G′, G″), complex viscosity (η*), and hardness. Biologically leavened doughs had a lower pH, influencing color, and swelling percentage, which is linked to the enrichment form and phenolic content. Extract-fortified doughs displayed increased G′, η*, and hardness compared to the control, whereas yeast-leavened doughs showed reduced swelling ability. Physicochemical changes were more significant in the yeast-leavened systems, which also scored higher on the sensory evaluations. Supplementing bakery products with bioactive fruit components enhances antioxidant status, but the enrichment form and proofing conditions significantly affect the physicochemical and sensory properties of the product.

## 1. Introduction

Nowadays, food functionality has become increasingly popular in the food industry. Bioactive compounds extracted from vegetable matrices have been used in product development to enhance the nutritional profile of basic food products, with respect to consumer health. Phenolic compounds are naturally occurring substances found in the plant kingdom, being more prominent in fruits and vegetables [1,2,3]. A well-documented role of phenolics has been demonstrated in the literature for the prevention of several diseases, shielding body tissues against oxidative stress, presenting benefits for gastrointestinal function and weight management, and exerting anticancer and antiglycation activities among other effects [1,4]. Bread and other bakery products are low-priced widely consumed foods that are currently used as matrices for the incorporation of fruit- and vegetable-derived phenolic compounds to improve their functional properties and health-promoting profile [4,5,6,7]. Nonetheless, bread production is a highly intricate process with numerous physical and biochemical factors potentially affecting the availability of bioactive compounds. Within this context, research has shown that the incorporation of grape pomace powder into wheat-based bread resulted in an increase in dough water absorption and stability, while a significant impact on the degree of softening was also established [6,8]. Moreover, [8] highlighted that phenolic compound incorporation in the wheat matrix may impact antioxidant activity and total phenolic content, along with the dough’s viscoelastic properties (gumminess, strength, adhesiveness, elasticity, and chewiness). The organoleptic properties of bread were also found to be dependent on phenolic compound addition, possibly due to the interaction between wheat protein and phenolic substances [8]. Enhancing our understanding of how process conditions and the interplay between bioactive compounds influence this can undoubtedly improve the functional qualities of enriched bread products [7].

Red fruits such as sweet cherry (*Prunus avium* L.) and sour cherry (*Prunus cerasus* L.) are rich sources of phenolic compounds; however, their year-round consumption is rather challenging due to their seasonality (only available during the summer months). Moreover, the majority of the crop is consumed as fresh fruit or paired with something sweet, such as yogurt or honey, to balance the flavor in the case of sour cherry. They may also be a refreshing addition to salads or a topping for various dishes. Secondary-grade or rejected fruits are mainly used to produce jams or alcoholic beverages. Within this context, there has been an increased interest in the valorization of this production and side-stream phenolic content to produce new functional products with potential health benefits [3,9,10,11].

Both sweet cherry and sour cherry are rich in sugars, organic acids, dietary fiber, minerals, vitamins, and antioxidants (polyphenols, anthocyanins, flavones, and flavonoids). The phytochemical composition of these fruits incorporates different types of antioxidant and phytochemical compounds such as carotenoids, polyphenols, including phenolic acids (hydroxybenzoic and hydroxycinnamic acids) and flavonoids (anthocyanins, flavonols, and flavan-3-ols), and Indolamines [3]. Until now, there are studies demonstrating that the presence of all these compounds could be transformed in products produced with heating processes, leading to higher phenolic content and antioxidant activity in the final product [12]. Organic acid content may vary from 2.9 g/L to 24.7 g/L depending on the species and cultivating conditions (microclimate, fertility, irrigation, etc.) [13,14]. The differences in the acidity of each variety may influence not only the color and taste of the fruit but also its physicochemical properties, leavening capability, and organoleptic properties, as well as the mechanical characteristics of the final product when incorporated in different matrices [13,14]. Several studies have highlighted the importance of fortifying foods using different sources of phenolic compounds from red fruit [15,16,17,18], which can be recovered with green extraction solvents (water/methanol and water/ethanol mixtures at different ratios that are generally considered as food-safe in product development [19,20,21]. Phenolic compounds obtained through different fruit or vegetable sources (mango seed kernel, orange peel, pomegranate seeds, grape marc, lettuce waste, broccoli stems or leaves, onion skin, and winery byproducts) have been found to increase the total phenolic content and antioxidant activity of bakery products [4,6]. Furthermore, the form (E., P.) of the ingredient incorporated has been reported to influence water interactions and bioactive compound retention during bread-making processes [7]. The most commonly used red fruits are those with medium acidity, such as strawberries, blackcurrants, raspberries, and their by-products (pomace, seeds, etc.) [9]. Gornas et al. showed that enrichment of muffins with red fruit (strawberry, sour cherry, raspberry, and black currant) pomace maintains the phenolic content at high levels in a high-temperature short-duration baking process [22]. Additionally, Cairone et al. underlined the perspective of using sour cherry phenolic compounds for the production of functional foods with added nutritional value [10].

To the best of our knowledge, there is limited information on the enrichment of bakery products using high-acidity fruits, such as sour cherry. Therefore, the aim of this study was to evaluate the effect of the incorporation of aqueous ethanol sour cherry extracts, in different forms, on the physicochemical properties of dough. The proposed strategy of this research can be used as a means for increasing the application of their use in a wide range of bakery products, providing added value for antioxidant-poor matrices and enhancing their nutritional and functional value. Moreover, a comparison between sour and sweet cherry extracts and their powder forms was performed in order to investigate the impact of pH, as a function of processing and leavening conditions, on the physicochemical and organoleptic properties of the bakery products.

## 2. Materials and Methods

### 2.1. Chemicals

Frozen cherry and sour cherry (Alterra, S.A., Giannitsa, Greece), dry yeast (Global Synergy Buying Group S.A., Sindos, Greece), sodium pyrophosphate/sodium bicarbonate (Jotis S.A., Athens, Greece), and all ingredients for making bread were purchased from the local market. The ethanol/water mixture used for the extraction of sweet cherry (*Prunus avium* L.) and sour cherry (*Prunus cerasus* L.) was obtained from local producers. Bis-(trimethylsilyl)-trifluoroacetamide (BSTFA), analytical grade ethanol, quercetin, 3-(4-hydroxyphenyl)-1-propanol, homovanillic acid, phloretic acid, oleanolic acid, cinnamic acid, vanillin, *p*-coumaric acid, chlorogenic acid, catechin, syringic acid, Folin–Ciocalteu reagent, and gallic acid were obtained from Sigma Chemical Co. (St. Louis, MO, USA). Tyrosol, protocatechuic acid, sinapic acid, *o*-coumaric acid, caffeic acid, and epicatechin were purchased from Fluka (Steinheim, Germany), vanillic acid was obtained from Serva (Heidelberg, Germany), and kaempferol, chrysin, naringenin, acacetin, and apigenin were obtained from Extrasynthèse (Genay-Cedex, France).

### 2.2. Total Phenolic Content

For the determination of the total phenolic content (TPC) of hydroalcoholic extract and fruit powder-based formulations, an adaptation of a previously reported protocol [23] was used, employing the Folin–Ciocalteu micro-assay. Gallic acid was used as a reference standard, and the results were expressed as mg gallic acid equivalents (GAE) per 100 g of lyophilized extract and pulverized fruit.

### 2.3. GC-MS Analysis of Phenolic Compounds

For the determination of simple polyphenols and triterpenic acids, aliquots of the extract (0.1 mL) were transferred to GC vials, an internal standard was added (3(4-hydroxyphenyl)-1-propanol solution, 19.2 μg/mL, 50 μL), the sample was evaporated to dryness under nitrogen, and derivatized by the addition of 250 μL BSTFA at 70 °C for 20 min. An aliquot (1 μL) of the derivatized sample was injected into the gas chromatograph at a split ratio of 1:20. GC-MS (Agilent, Wallborn, Germany) coupled with an HP 5973 MS detector, split—splitless, injector, and an HP 7683 autosampler by employing an HP-5 MS capillary column (5% phenyl–95% methyl siloxane, 30 m 0.25 mm 250 μm lm) were used for the determination of the initial phenolic compound content, present in sour cherry and cherry lyophilized powder and lyophilized extract. A selective ion-monitoring GC-MS method was applied for the determination of 29 compounds, as previously described by [24]. The detection of the compounds was based on the ±0.05 RT presence of target (T) and qualifier ions of the standard polyphenols and triterpenic acids at the predetermined ratios. Identification of chromatographic peaks was achieved by comparing the retention times and ratios of two or three fragment ions of each phenolic compound with those of reference compounds, while quantification was carried out by employing 3-(4-hydroxyphenyl)-1-propanol as an internal standard.

### 2.4. Preparation of Enrichment Formulations and Making the Bread

Phytochemical-rich extracts of sour cherry (*Prunus cerasus* L) (S.Ch.) and cherry (*Prunus serotina*) (Ch.) were obtained according to a previously described method [5]. A green extraction procedure, optimized through preliminary experiments, was used for the preparation of aqueous ethanol extracts. Specifically, aqueous ethanol extracts were prepared using 3 mL of solvent (water/ethanol 1:1, *v*/*v*) and 0.5 g lyophilized pulverized red fruits (sweet cherry (*Prunus avium* L.) and sour cherry (*Prunus cerasus* L.)) (E.), with periodical stirring at room temperature for 24 h. The prepared extracts were further subjected to evaporation under vacuum for the removal of ethanol followed by lyophilization (D.E.). Fruit powder (P.) was prepared via pulverization of lyophilized red fruits, which were passed through a 2 mm sieve to ensure the uniform size of powder particles. Five bread formulations were prepared for each fruit using different phytochemical-rich preparations, E.: Extract (30% fruit content); D.E.: Dehydrated Extract (2% fruit content); P.: Pulverized Fruit (3% fruit content); E./P.: Extract combined with pulverized fruit (29%/3% fruit content); and D.E./P.: Dehydrated Extract combined with pulverized fruit (2%/3% fruit content). A control sample (C.), which did not contain phytochemical-rich preparations, was also prepared. The initial TPC of the hydroalcoholic extract and the lyophilized pulverized fruit was: 4.0 ± 0.6 mg/mL and 189.1 ± 32.6 mg/100 g dry fruit for sour cherry and 0.7 ± 0.1 mg/mL and 31.8 ± 5.7 mg/100 g dry fruit for cherry, respectively. The remaining materials for the prepared bread formulations were wheat flour (51–54%), sugar (7%), salt (1%), olive oil (7%), and water (Table A1). Preliminary experiments were performed to determine the amount of water replaced by the extract with respect to the dough structure.

Two raising agents were used for each trial, baking powder (sodium pyrophosphate/sodium bicarbonate (chemical leavening)) and Saccharomyces cerevisiae (biological leavening). Control samples (blank formulations), without the incorporation of phytochemical-rich compounds, were also prepared. Bread-making, proofing, and baking process were performed as previously described [5]. 

### 2.5. Swelling Capacity (%) of Dough

To evaluate the development of dough swelling as a function of leavening agents and the incorporation of sweet and sour cherry phytochemical-rich preparations, dough samples were measured using a Vernier caliper. Dimensional changes were measured on dough samples molded in small spheres of 40 g each, after the fermentation process at 40 °C (15 min for baking powder samples, 60 min for yeast samples). Equations (1) and (2) were used for the determination of dough sphere volume and % swelling:

(1) $$V_{sphere}=\frac{4}{3}\pi r^{3}$$

(2) $$\%Swelling=\frac{V_{final}-V_{initial}}{V_{initial}}\times 100$$

### 2.6. Physicochemical Properties

The water activity of dough samples was measured using a water activity meter (Hygropalm 23, Rotronic, West Sussex, UK) at 25 °C. The official AOAC method was used for the dough’s moisture analysis. Briefly, dough samples (approximately 2 g) were weighed and placed in a laboratory oven at 105 °C for 24 h. All analyses were performed in triplicate.

Dough and bread pH were measured using a pH meter (SI Analytics Lab 845, Xylem Inc., Washington, DC, USA) at room temperature. An appropriate amount of the sample was weighed in a dry sample beaker, and free water was added. The sample was homogenized under magnetic stirring for 30 min. For pH determination, the supernatant was carefully decanted, and the pH was measured immediately.

### 2.7. Color Analysis

Color analysis of the dough, bread crumb, crust, and their mixtures was assessed spectrophotometrically (CM-5, Konica Minolta, Osaka, Japan). The color parameters L* (100 =white, 0 = black), a* (−value = green, + value = red), and b* (−values = blue, + values = yellow) were measured by placing an appropriate quantity of well-homogenized dough or graded bread. A black plate (supplied by the manufacturer) was used as the standard.

The total color difference (ΔΕ) was defined by the Minolta Equation (3) [25]:

(3) $$\Delta Ε=\sqrt{\Delta L^{2}+\Delta a^{2}+\Delta b^{2}}$$

where:

$\Delta L={L^{*}}_{control\;dough}-{L^{*}}_{dough\;with\;BC}$**,**
$\Delta \alpha ={a^{*}}_{control\;dough}-{\alpha^{*}}_{dough\;with\;BC}$**,**
and $\Delta b={b^{*}}_{control\;dough}-{b^{*}}_{dough\;with\;BC}$.

### 2.8. Viscoelastic Properties of Dough

Small-amplitude oscillatory shear measurements were performed in control and enriched doughs using a stress-controlled rheometer (MCR 102, Anton Paar, GmbH, Graz, Austria), at 22 °C, with a parallel plate geometry of 25 mm and a 2 mm gap. To determine the Linear Viscoelastic Range (L.V.R) of the samples, amplitude sweeps were performed, and the shear strain was set at γ = 0.05% (Figure A3). Frequency sweep measurements were obtained for all samples, where Storage Modulus (G′) and Loss Modulus (G″), representing the elastic and viscous properties of the material, alongside the phase angle δ (tanδ, G″/G′, representing the phase difference between stress and strain during oscillation) and Complex Viscosity (η*, Pa* s, describing the frequency dependence of viscosity), were recorded and analyzed as a function of Angular Frequency (ω, 0.1–100 rad/s) with RheoCompass software (Version 1.15). All measurements were performed at least in triplicate.

### 2.9. Texture Analysis of Dough and Baked Bread 

Texture profile analysis of the dough and bread crumb of control and enriched samples was performed using a texture analyzer (TA.HDplus, Stable Micro Systems, Godalming, Surrey, UK), as previously described [5]. Texture parameters of hardness, cohesiveness, and gumminess were analyzed using the Exponent Connect v7.0 software.

### 2.10. Sensory Analysis

For the organoleptic evaluation of the prepared enriched breads, two different types of tests were conducted with the participation of 10 well-trained sensory panelists (two men and eight women) recruited from the staff and students of the Department of Nutrition and Dietetics of Harokopion University of Athens. Panel orientation consisted of 2 h of product-specific attribute/definition development and the exploration of appropriate product references. Scoring on a 5-point Hedonic scale (1—Disliked, 2—Slightly disliked, 3—Neither Liked nor Disliked, 4—Slightly liked, 5—Liked) was used to determine the taste, texture, color, aroma, and overall acceptance of the enriched bakery products. First, 2 ranges of 6 samples (including the control) were performed to assess panelists’ preferences based on the type of leavening agent and the type of fruit used for the enrichment. Control samples were prepared and tested to determine the optimum leavening agent. Panelists evaluated texture attributes using a piece of bread of approximately 5 cm^2^. All possible combinations were prepared to evaluate the optimum type of enrichment for each fruit. To determine the overall fruit preference in bread formulations, (Ch. vs. S.Ch.), an additional sensory assessment of 4 samples was carried out, where the most desirable enriched sample from each fruit was comparatively selected. Samples were evaluated under white LED lights to determine the color acceptance.

### 2.11. Statistical Analyses

Data handling was performed using Microsoft Excel (Microsoft Corporation, Redmond, WA, USA). Values are the mean of triplicate measurements (*n* = 3). Statistical analyses were performed using SPSS software (SPSS 20.0 for Windows, Chicago, IL, USA) and one-way ANOVA. Duncan’s multiple range tests were performed post hoc to evaluate differences between groups. The significance level was set at 0.05, and the independent sample *t*-test was used to evaluate any significant differences between groups, which were determined based on the raising agents used. 

## 3. Results

### 3.1. Impact of Enrichment on the Dough’s Physicochemical Properties

**Figure 1:**
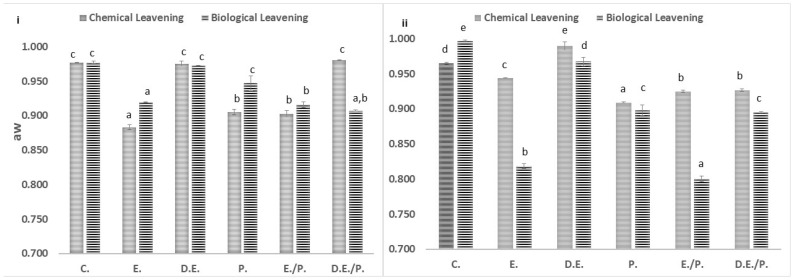
Water activity of doughs. Data are presented as the average values of three replications ± standard deviation. Lowercase letters indicate significant differences (*p* < 0.05) at a 95% confidence level. (**i**) Cherry; (**ii**) S. Cherry; C.: Control sample; E.: Extract; D.E.: Dehydrated Extract; P.: Pulverized Fruit; E./P.: Extract combined with pulverized fruit; D.E./P.: Dehydrated Extract combined with pulverized fruit.

The physicochemical properties of dough samples enriched with red fruit phytochemical-rich formulations are given in Table 1. As shown in this Table, the two sources (Ch. and S.Ch.) of bioactive phytochemicals displayed different interaction patterns in terms of color variation (ΔE), pH, and total phenolic content in the same matrix (wheat-based dough). These differences were based on the enrichment forms’ chemical composition (TPC, pH, and ethanol content) and the origin of the fruit (Ch. and S.Ch.). The initial phenolic content and various enrichment forms (extract, dehydrated extract, pulverized fruit, extract combined with pulverized fruit, and dehydrated extract combined with pulverized fruit) presented significant differences between fruits (Table A2). The prevalence of S.Ch. in terms of higher TPC was exhibited in all types of different enrichment forms applied, as shown in Table 1. Figure 1 presents the color variation.

The brightness (L*) and color difference (ΔΕ) of the samples were statistically correlated with the total phenolics added, as shown in Table A3 (Appendix A). Supplemented phenolic content was significantly related to chromatic parameters (L*, a*, and b*) and color difference (*p* < 0.05). Products with higher phenolic potential (E./P., D.E./P., and D.E.) exhibited higher ΔΕ values, except for the ones containing cherry fruit powder. Contrary to this observation, sour cherry presented lower ΔΕ values for all types of enrichment, attributed to its natural acidity [3].

#### 3.1.1. Dough Water Activity

While the water activity (aw) of the doughs was relatively stable across almost all trials, a noteworthy decrease was observed in the samples containing an extract as an enrichment agent (Figure 1). Samples without the addition of bioactive microconstituents and instant yeast as leavening agents had higher aw values [5]. Furthermore, doughs of both fruits enriched with dehydrated fruit exhibited reduced water activity (aw), possibly due to the higher levels of fiber and sugars compared to the ones enriched with extract and control samples [2,3,11].

#### 3.1.2. Swelling Capacity

**Figure 2:**
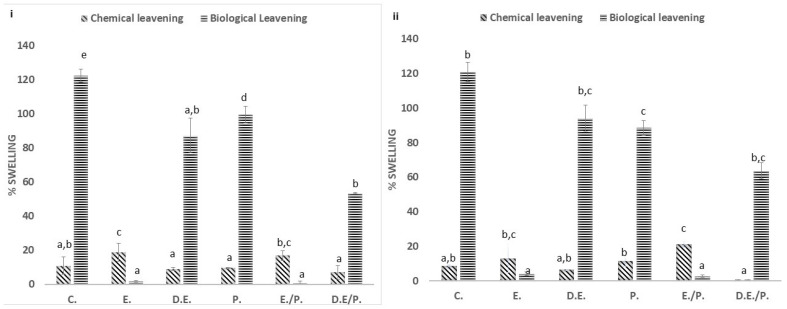
Swelling Capacity (%). Data are presented as the average values of three replications ± standard deviation. Lowercase letters indicate significant differences (*p* < 0.05) at a 95% confidence level. (**i**) Cherry; (**ii**) S. Cherry; C.: Control sample; E.: Extract; D.E.: Dehydrated Extract; P.: Pulverized Fruit; E./P.: Extract combined with pulverized fruit; D.E./P.: Dehydrated Extract combined with pulverized fruit.

Two distinct types of leavening agents, biological and chemical, have been employed for swelling. The expansion capacity (% Swelling) of dough displayed statistically significant differences between the control and enriched samples, as well as between the different enrichment methods. For samples enriched with extracts (either individually or in combination with pulverized fruit), no significant expansion capacity was observed when instant yeast was used as the leavening agent. Conversely, when a chemical reagent was used as the leavening agent, the same samples exhibited a higher degree of expansion capacity than those with biological leavening. When comparing the two leavening agents, biological leavening led to greater swelling than chemical agents. As illustrated in Figure 2, swelling capacity displayed the following increasing order: chemical leavening < biological leavening; and extract < extract combined with pulverized fruit < dehydrated extract combined with pulverized fruit < dehydrated extract = pulverized fruit < control. 

Samples with higher sugar content (D.E/P. and D.E.) showed greater expansion capacity compared to other enrichment types, irrespective of the type of fruit used. However, compared with sour cherry extract-enhanced dough, cherry extract dough displayed a higher ΔE. This can be explained by the structure of the extract, which is more acidic in the case of the sour cherry than that of the cherry (pH values of 3.6 and 3.2, respectively). In this study, there was no notable distinction in swelling capacity between the two fruits in all types of enrichment. 

### 3.2. Individual Phenolic Compounds

As mentioned in the literature, the two fruits studied present differences with respect to their total phenolic content (TPC) and phenolic profile (Figure A2), which may influence not only their antioxidant capacity but also their physicochemical properties and their acceptance by consumers [2,9,10,11]. In order to evaluate the phenolic content of each fruit, each form of enrichment was evaluated in terms of its phenolic profile. 

**Figure A1:**
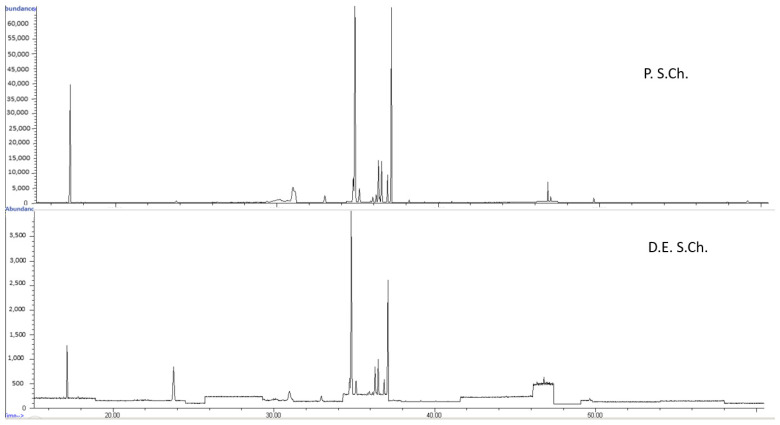
Representative GC-MS chromatograph of two different forms of enrichment (P. and D.E.) for sour cherry fruit (S.Ch.).

**Figure A2:**
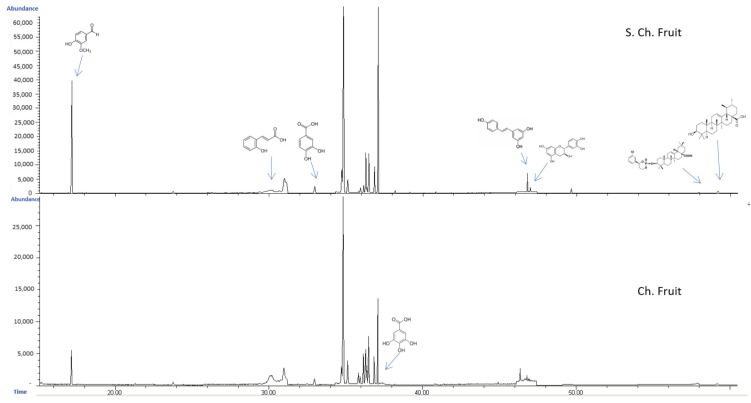
GC-MS chromatograph for cherry (Ch.) vs. sour cherry (S. Ch.) pulverized fruit.

**Figure A3:**
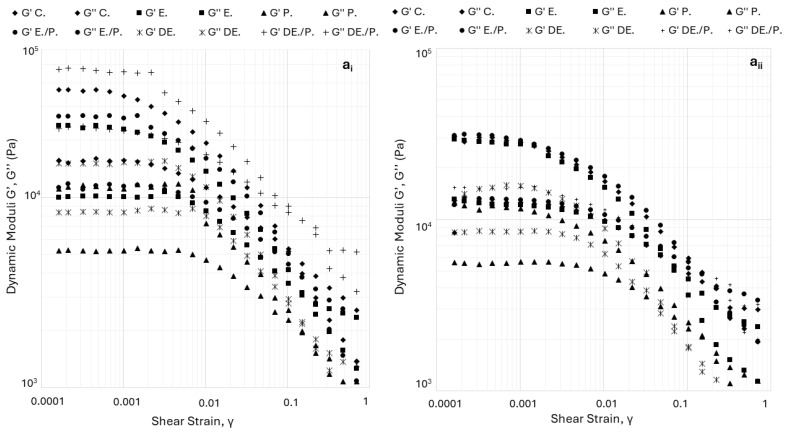

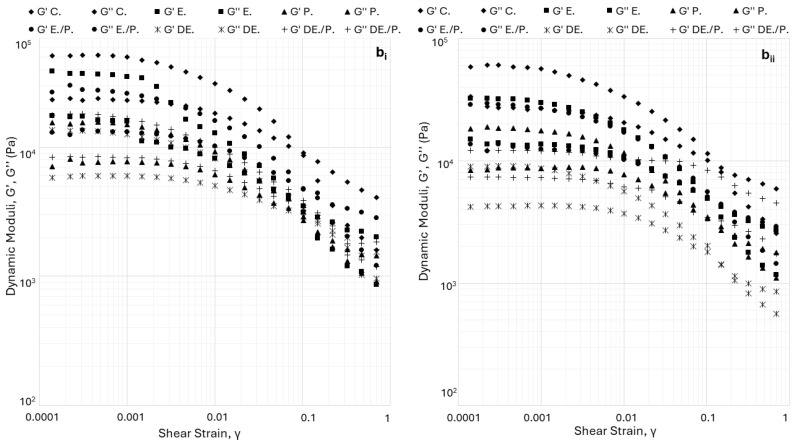
Amplitude sweep diagram for (**ai**) Cherry-enriched doughs prepared with chemical leavening and (**aii**) Cherry-enriched doughs prepared with chemical leavening; (**bi**) Sour Cherry-enriched doughs prepared with chemical leavening and (**bii**) Sour Cherry-enriched doughs prepared with chemical leavening.

Phenolic compounds found in sour cherry and cherry fruit extracts are given in Table 2. The content of sour cherry in identified and quantified phenolic compounds was higher than that of cherry, in line with the literature [3,11,26]. Differences were observed between the pulverized fruits and the extracts of both fruits (Figure A1). Sour cherry fruit presented higher contents for the majority of the phenolic compounds analyzed, namely cinnamic acid, ursolic acid, protocatechuic acid, vanillin, epicatechin, quercetin, catechin, chrysin, ferulic acid, and kaempferol. Lyophilized cherry powder contained higher levels of oleanolic acid, gallic acid, caffeic acid, p-hydroxybenzoic acid, vanillic acid, and ferulic acid than cherry. The aforementioned compounds were identified in both matrices (S.Ch. and Ch.) and accounted for 3% to >50% of the identified phenolic content, with a substantially higher percentage being presented in sour cherry pulverized fruit for Tyrosol, p-hydroxybenzoic acid, phloretic acid, vallinic acid, syringic acid, sinapic acid, oleanolic acid, ursolic acid, and kaempferol.

### 3.3. Rheology

**Figure 3:**
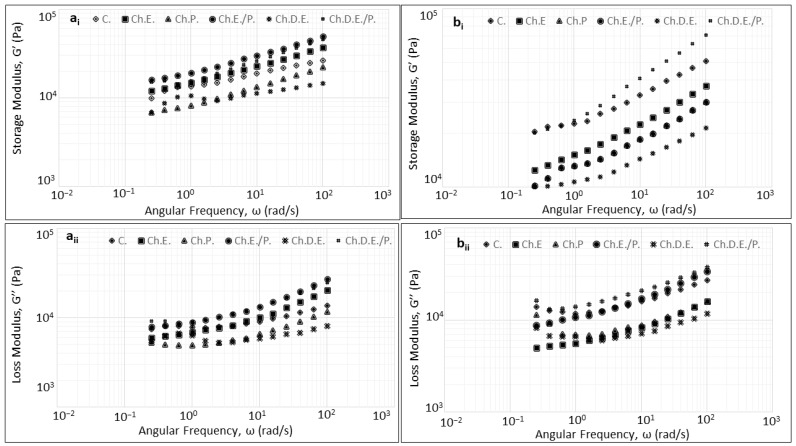
Impact of the addition of enrichment agents based on cherry extract and powder forms on (**a_i_**) Storage Modulus and (**a_ii_**) Loss Modulus for chemically leavened doughs and (**b_i_**) Storage Modulus and (**b_ii_**) Loss Modulus for chemically leavened doughs.

**Figure 4:**
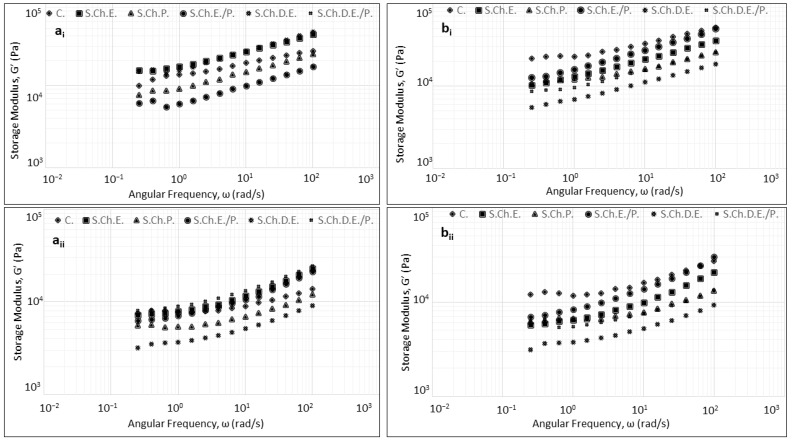
Impact of the addition of enrichment agents based on sour cherry extract and powder forms on (**a_i_**) Storage Modulus and (**a_ii_**) Loss Modulus for chemically leavened doughs and (**b_i_**) Storage Modulus and (**b_ii_**) Loss Modulus for chemically leavened dough.

The viscoelastic properties of wheat doughs prepared with cherry and sour cherry enrichment agents are shown in Figure 3 and Figure 4. For all samples assessed under both proofing conditions, wheat dough displayed typical behavior as a highly structured material with a viscoelastic response, which was expressed by the predominance of the elastic over viscous modulus (G′ > G″) in the studied frequency range [27,28]. This behavior characterizes the highly elastic properties of the dough, which are indicative of the gluten network that behaves like a crosslinked polymer with hydration [29]. Moreover, for all studied samples, both moduli (G′ and G″) exhibited a rising trend with increasing frequency (0.1–100 rad/s), while tanδ values were less similar, suggesting weak gel behavior, which is well-described in wheat dough systems [30]. In cherry-enriched doughs formulated with chemical reagents, higher values of both moduli were established compared to fermented doughs. In fermented cherry dough samples, enrichment with E., E./P., and D.E./P. was shown to increase the dynamic moduli with respect to the control formulation, suggesting a denser structure. On the contrary, the addition of P. and D.E. to fermented samples was found to decrease G′ and G″ values, being more pronounced in formulations prepared with D.E. This behavior is probably related to the high sugar content of cherry fruits [31]. In this context, chemically formulated doughs displayed similar results regarding the dynamic moduli with the addition of D.E./P., inducing the greatest increase in G′ and G″ values. 

In sour cherry dough samples prepared with a chemical reagent, the dynamic moduli also displayed increased values compared to fermented doughs, with the exception of E. and D.E. samples. Furthermore, the addition of enrichment agents to dough resulted in a decrease in both moduli with respect to the control formulation. 

### 3.4. Texture

The results of texture profile analysis performed on doughs prepared with phytochemically rich cherry and sour cherry forms as a function of different proofing conditions are displayed in Table 3. In the case of cherry-enriched doughs, significant differences (*p* < 0.05) in hardness were observed compared to the control sample. Specifically, for the samples fermented with E., E./P., and D.E./P. with cherry incorporation, a notable increase in dough hardness was observed, whereas P. and D.E. incorporation did not differ significantly from the control sample.

The results concerning the parameter of cohesiveness result in the same pattern since Ch.E., Ch.E./P., and Ch.D.E./P. doughs were significantly less cohesive than the control sample, suggesting a lesser ability of the dough to withstand deformation, while gumminess, as the calculated product of hardness multiplied by cohesiveness, also produced similar results. For the chemical leavening agent (baking powder), hardness and cohesiveness did not display the same trend as the yeast-treated formulations. Specifically, the pulverized fruit (Ch. P.) and dehydrated extract and pulverized fruit (Ch. D.E./P.) were shown to induce a hardening effect in dough, decreasing its cohesiveness. This can be attributed to the differences in pH between the two proofing methods.

In addition, sour cherry-supplemented dough displayed similar results in terms of hardness, cohesiveness, and gumminess when the extract was added. With respect to the baked bakery product, as shown in Table 4, fermented and chemically leavened cherry and sour cherry products exhibited the same trend as in dough: increased values of hardness and gumminess, and reduced cohesiveness when extract was added.

### 3.5. Organoleptic Evaluation

**Figure 5:**
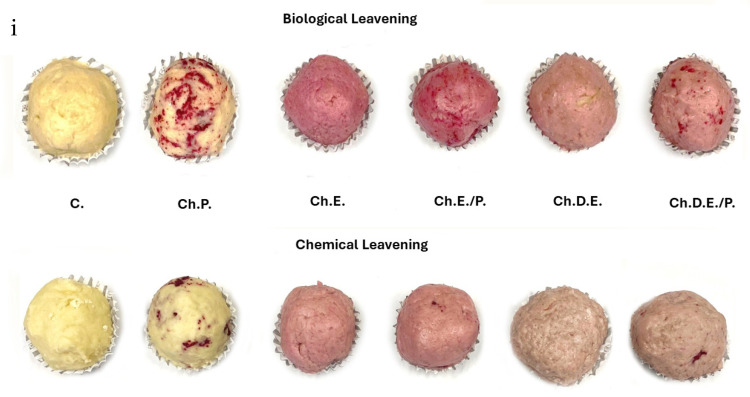

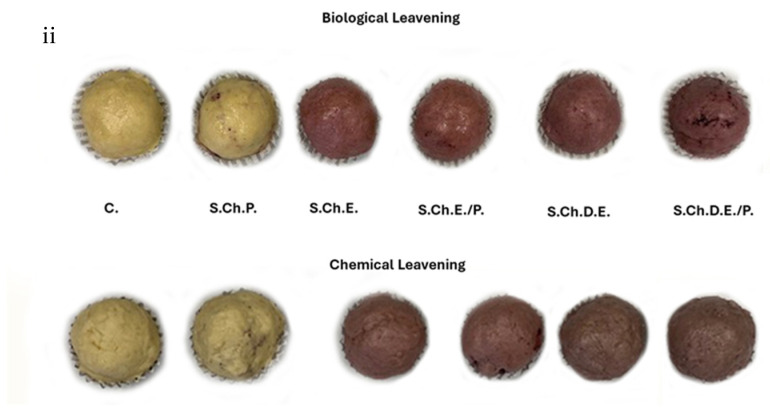
Color variations for (**i**) control and enriched doughs based on various forms of cherry extract and powder and (**ii**) control and enriched doughs based on various forms of sour cherry extract and powder for two different proofing environments (biological and chemical leavening).

**Figure 6:**
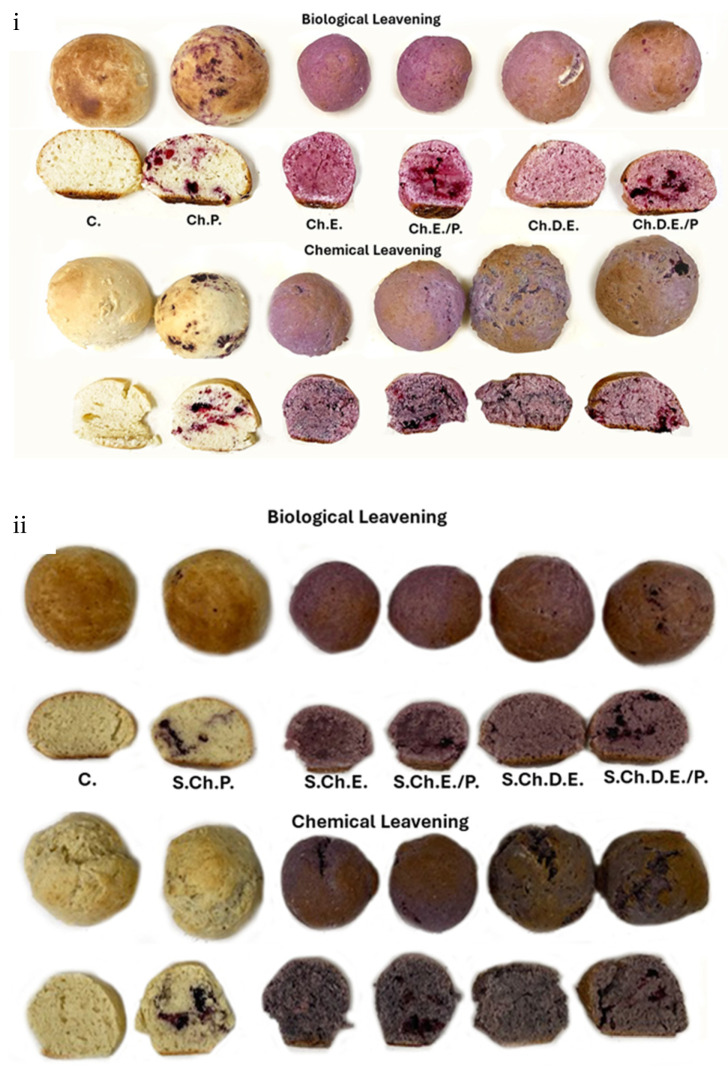
Color and texture variations for (**i**) control and enriched breads based on various forms of cherry extract and powder and (**ii**) control and enriched breads based on various forms of sour cherry extract and powder for two different proofing environments (biological and chemical leavening).

**Figure 7:**
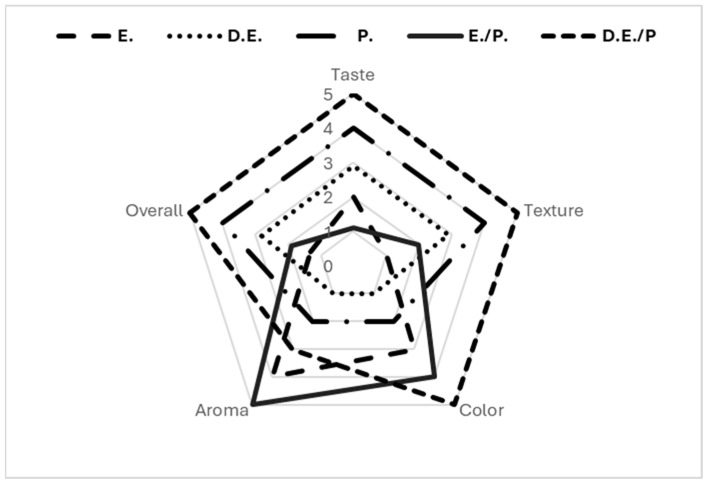
Organoleptic evaluation of enriched breads. Data are presented as the average values. C.: Control sample; E.: Extract; D.E.: Dehydrated Extract; P.: Pulverized Fruit; E./P.: Extract combined with pulverized fruit; D.E./P.: Dehydrated Extract combined with pulverized fruit.

It was generally accepted that breads prepared with Saccharomyces cerevisiae (biologically leavened) were more preferred than those with sodium bicarbonate (*p* = 0.05). Sample comparison with respect to different types of enrichment highlighted that even if aroma and color were found to be more intense for the samples enriched with extracts (Figure 5 and Figure 6), the trained panelists better preferred those prepared with powder forms (dehydrated extract and pulverized fruit) because of their texture and the absence of an ethanol taste, as demonstrated in Figure 7. As shown in Figure 5 and Figure 6, samples with cherry were lighter than samples with sour cherry and the chemical leavened samples presented a less vivid color. Overall, the most preferred samples were those with dehydrated extract combined with the pulverized fruit, as well as those with pulverized fruits (*p* = 0.05). Finally, no significant differences were noticed when comparing sour cherry- and cherry-enriched bakery products. However, there was a trend for preferring cherry over sour cherry (60% of the testers preferred cherry as a fortification agent over sour cherry), possibly due to its mild acidity.

## 4. Discussion

Color variations (ΔΕ) were significantly affected by the enrichment type, the leavening environment, and the phenolic content added. The observed differences between the two different fruits (cherry and sour cherry) may be attributed to the extract’s origin, which is more acidic in the case of sour cherry compared to cherry. Differences in TPC were observed between sweet and sour cherry, as has already been described [2,11,26]. Moreover, the variations of TPC between the different types of enrichment could also be attributed to other components (fibers, sugars. etc.) that are either absent in the extract (and dehydrated extract) or present in much lower quantities (indicating that they have been largely removed during the extraction process).

At the same time, color variations could be influenced by leavening conditions. Leavening agents can generate carbon dioxide through two mechanisms: chemical reactions and microbial fermentation (instant yeast; *S. cerevisiae*). When chemical leavening agents are incorporated, they speed up the fermentation process by inducing a rapid expansion capacity. This acceleration is primarily a result of the chemical interaction between these agents, notably sodium pyrophosphate and sodium bicarbonate, and the acidic conditions within the mixture. Consequently, the utilization of chemical leavening agents leads to the neutralization of the acidity introduced by the fruit or fruit extract, resulting in a shift towards a less acidic pH.

A decrease in water activity was observed for both fruits with the addition of extract-based formulations. This finding aligns with the research of Miskiewicz et al., who suggested that the presence of the extract enhances the water-binding capacity of the dough ingredients [32,33] Moreover, the presence of a small amount of ethanol in the extract replaced the total quantity of water added during the dough formulation, which subsequently increased water evaporation during proofing because of the azeotropic mixture formed between water and ethanol.

The swelling capacity of doughs was found to differ significantly both for the proofing environment and enrichment type. Chemical leavening agents play a vital role in various technological functions that influence dough handling and the quality of the final product, encompassing sensory and leavening properties [34] Alcohol present in extract enrichment forms, while impacting *S. cerevisiae* adversely by inhibiting microorganism growth, does not significantly impact this function in the case of chemical reagents. Multiple studies indicate that the specific volume decreases when extracts from sources rich in phenolic compounds (such as agricultural by-products) are added, even when the extract is water-based [35,36]. Consequently, enriching bread with these extracts notably reduces the specific volume. This effect can be mitigated by the acidifying impact of these additives, which can alter the gluten network structure [37].

The addition of bioactive ingredients can physically alter the bread dough’s development by forming complexes with proteins and/or polysaccharides. These reactions involve hydrogen bonding between phenolic compound hydroxyl groups and the peptide residue carbonyl groups of proteins, leading to stable complexes via covalent and ionic bonds. Additionally, complexation can involve hydrophobic interactions or cross-linking between polyphenols and proteins or among different protein molecules [35,36].

GC-MS analysis performed on the two different fruit types presented significant variations in terms of the phenolic content; these were dependent on the fruit type as well as the enrichment form. The compounds found in the extract were typically lower in content than that of the corresponding fruit, given that during the green extraction process, a significant portion of phenolic compounds is lost. When comparing extracts to fruit powder, it was evident that for both fruits, approximately 50% of compounds were absent and their quantities were reduced by up to 60 times. Sour cherry enrichment forms were found to be more pronounced with respect to their phytochemical content in comparison to cherry forms. These findings highlight sour cherry as a highly potent enrichment agent with a possible beneficial role in quality parameters and a health-promoting profile for gluten-free product development.

Dynamic oscillation measurement is a reliable tool for studying the viscoelastic properties of doughs subjected to varying degrees of deformation. Thus, valuable information can be generated about the dough’s microstructural attributes and physical structure as a function of its chemical composition [38]. The investigation of viscoelastic properties in terms of the leavening environment for doughs prepared with cherry-based enrichment forms presented an increment of dynamic moduli in chemically leavened doughs compared to doughs subjected to fermentation. These results suggest a stiffer structure formation induced by pH differences due to different proofing environments. This behavior was also highlighted in a previous study [39], where an increase in alkali concentration led to an increase in dough elasticity, enhancing the formation of the dough’s protein network [40].

The addition of extract-based cherry formulations was shown to increase dynamic moduli values in comparison with blank formulations (Control). This behavior is possibly an aftereffect of the azeotropic mixture formation between water and ethanol that further reduces moisture and water activity values in samples prepared with extract addition. This dependency of dynamic moduli on water content has been reported in the literature with an increase in storage and low modulus values when water content decreases [40]. Regarding the impact of sour cherry addition on the viscoelastic properties of dough, a decrease in dynamic moduli was observed with respect to control formulation, being more pronounced in chemically leavened products, while a significant decrease in dough pH values in both leavening conditions was also evident. This phenomenon can be attributed to the rich profile of sour cherry fruit in organic acids, which has been reported to negatively interfere with the dough’s cohesiveness [31,41].

The texture attributes of enriched doughs displayed variations between different enrichment types, where extract-based formulations were shown to increase hardness in both fruits. Similar results were also observed by the authors in a previous published study regarding the enrichment of bakery products with grape-derived phytochemicals [5]. The parameter of hardness in doughs is typically associated with limited water distribution within the food matrix, as well as the ability of specific ingredients, such as fiber, to bind water molecules [42,43]. Therefore, the addition of aqueous ethanol extracts in a wheat matrix possibly acts as a limiting factor for water activity and consequent proper dough hydration, resulting in a stiffer dough texture. This fact can be further substantiated by water activity and moisture content results. The parameter of cohesiveness is representative of the sample’s resistance to deformation before breaking, which can characterize the internal bond strength within a food matrix [44,45]. The addition of an aqueous ethanolic extract resulted in a reduction in cohesiveness values, which has been reported to induce the solubilization of gluten protein fractions [45,46]. Hence, upon solubilization, better swelling and expansion can be anticipated for the gluten network, which can subsequently result in the reduced cohesiveness of the dough system as a result of a decrease in the strength and amount of intermolecular secondary protein structures [44].

With respect to the impact of the addition of different enrichment forms based on cherry and sour cherry fruit on the textural attributes of baked breads, the same tendencies as in doughs were established, with an increase in hardness and reduced cohesiveness values. The cohesiveness in baked samples can be related to the moisture content and the network strength that surrounds the crumb cells, with lower cohesiveness values being associated with bread crumbling [44]. Therefore, it can be established that the addition of aqueous ethanol extracts in wheat dough matrices, which overall do not exceed the ethanol levels released during fermentation, inhibits yeast activity and can significantly impact the dough’s texture, causing stiffer, harder-to-process doughs with a higher tendency to crumble [45].

## 5. Conclusions

This study analyzed the effects of different dough enrichment methods with cherry and sour cherry, focusing on texture, color, and sensory preferences. Chemical enrichment generally led to stiffer and harder dough, notably with increased moduli. Heterogeneous effects were observed on dough cohesion and hardness, with some enrichment causing a decrease in these properties in both cherry and sour cherry doughs. Regarding the effect in the chroma, enrichment significantly impacted dough color, with variations observed between cherry and sour cherry samples. The expansion capacity was influenced by the enrichment method and leavening agent, with chemical agents favoring greater expansion compared to extracts. Moreover, the phenolic compounds added during enrichment correlated with color differences, with higher phenolic potential leading to increased color variation. Sensory preferences demonstrated that samples enriched with extracts were noted for intense aroma and color, whereas pulverized fruit forms were preferred for their texture and lack of an ethanol taste. The bread prepared with traditional yeast was generally favored over sodium bicarbonate leavening, pointing to a preference for traditional fermentation methods. In conclusion, the combinations of dehydrated extract and pulverized fruit, as well as products with only pulverized fruits, were most preferred, indicating a balance between flavor, texture, and visual appeal. No significant preference was found between cherry and sour cherry products, although a slight tendency towards cherry was noted due to its mild acidity.

According to the results of this study regarding viscoelastic, organoleptic, and physicochemical parameters, it was found that the application of high-quality secondary raw materials could be incorporated into the food industry in order to decrease the nutritional value of bread. This indicates that the introduction of secondary products of fruit crops in a powder form has a significant effect on the acceptance of the final product without influencing the process of bread preparation.

## Figures and Tables

**Table 1:** Physicochemical parameters (ΔΕ, pH) and supplemented total phenolics (TP, mg/100 g dough) of dough in different enrichment forms prepared using sour cherry and cherry fruits.

		Cherry Dough			Sour Cherry Dough		
		ΔE	pH	TP	ΔE	pH	TP
C.	Chemical Leavening	-	7.13 ± 0.06 ^b^		-	7.07 ± 0.00 ^e^	-
E.		32.99 ± 0.30 ^d^	6.92 ± 0.09 ^a,b^	95.2 ± 18.0 ^b^	24.88 ± 0.14 ^d^	6.42 ± 0.02 ^c^	123.8 ± 14.2 ^b^
D.E.		23.54 ± 0.03 ^b^	6.98 ± 0.07 ^a,b^	3.1 ± 0.4 ^a^	17.79 ± 0.19 ^b^	6.70 ± 0.05 ^b^	3.8 ± 0.7 ^a^
P.		28.75 ± 0.33 ^c^	6.94 ± 0.05 ^a^	95.2 ± 17.4 ^b^	23.49 ± 0.13 ^c^	6.11 ± 0.19 ^d^	123.4 ± 14.6 ^b^
E./P.		40.96 ± 0.31 ^f^	6.78 ± 0.06 ^b,c^	95.2 ± 18.0 ^b^	39.50 ± 0.09 ^f^	5.25 ± 0.04 ^a^	123.8 ± 14.2 ^b^
D.E./P.		36.55 ± 0.33 ^e^	6.80 ± 0.09 ^a^	98.4 ± 18.0 ^b^	38.04 ± 0.16 ^e^	5.53 ± 0.06 ^b^	127.6 ± 14.7 ^b^
C.	Biological Leavening	-	5.65 ± 0.06 ^d^	-	-	5.52 ± 0.01 ^c^	-
E.		31.84 ± 0.01 ^d^	5.73 ± 0.04 ^d^	95.2 ± 18.0 ^b^	30.27 ± 0.07 ^c^	4.68 ± 0.03 ^d^	123.8 ± 14.2 ^b^
D.E.		24.77 ± 0.03 ^c^	5.37 ± 0.15 ^b^	3.1 ± 0.4 ^a^	17.35 ± 0.08 ^b^	4.83 ± 0.01 ^a,b^	3.8 ± 0.7 ^a^
P.		24.15 ± 0.03 ^b^	5.17 ± 0.09 ^b^	95.2 ± 17.4 ^b^	31.11 ± 0.14 ^d^	4.34 ± 0.03 ^b^	123.4 ± 14.6 ^b^
E./P.		41.40 ± 0.16 ^f^	5.18 ± 0.01 ^c^	95.2 ± 18.0 ^b^	42.91 ± 0.12 ^f^	4.01 ± 0.03 ^a,b^	123.8± 14.2 ^b^
D.E./P.		38.90 ± 0.21 ^e^	4.97 ± 0.03 ^a^	98.4 ± 18.0 ^b^	36.89 ± 0.13 ^e^	4.31 ± 0.07 ^a^	127.6 ± 14.7 ^b^

Data are presented as the average values of three replications ± standard deviation. Lowercase letters indicate significant differences (*p* < 0.05) between samples at a 95% confidence level. C.: Control sample; E.: Extract; D.E.: Dehydrated Extract; P.: Pulverized Fruit; E./P.: Extract combined with pulverized fruit; D.E./P.: Dehydrated Extract combined with pulverized fruit.

**Table 2:** Phenolic compounds (mg/100 g d.w.) present in the pulverized fruit (P.) and dehydrated extract (D.E.) of cherry (Ch.) and sour cherry (S.Ch.) fruits.

	P. Ch.	P. S.Ch.	D.E. S.Ch.	D.E. Ch.
Cinnamic acid	149.52 ± 2.58	730.63 ± 15.23	17.98 ± 1.28	4.16 ± 0.97
Ursolic acid	58.65 ± 1.24	88.96 ± 9.18	n.d.	n.d.
Protocatechuic acid	47.52 ± 0.67	68.19 ± 2.35	1.28 ± 0.98	3.03 ± 0.25
Oleanolic acid	72.96 ± 4.83	15.54 ± 2.41	n.d.	1.32 ± 0.06
Gallic acid	57.81 ± 4.32	0.30 ± 0.07	0.25 ± 0.08	0.09 ± 0.01
Vanillin	10.78 ± 1.98	45.69 ± 2.35	1.17 ± 0.18	0.30 ± 0.06
Epicatechin	10.37 ± 0.25	51.32 ± 4.32	1.15 ± 0.11	n.d.
Quercetin	3.02 ± 0.18	4.66 ± 0.99	1.46 ± 0.09	1.67 ± 0.85
Caffeic acid	1.15 ± 0.04	0.74 ± 0.09	0.10 ± 0.02	0.06 ± 0.00
p-hydroxybenzoic acid	2.18 ± 0.11	0.49 ± 0.08	n.d.	0.32 ± 0.02
Vannilic acid	2.14 ± 0.14	0.77 ± 0.06	n.d.	0.25 ± 0.09
Catechin	2.28 ± 0.08	6.88 ± 0.45	0.19 ± 0.02	n.d.
Chrysic	2.24 ± 0.16	3.15 ± 0.94	1.83 ± 0.05	n.d.
Ferulic acid	0.37 ± 0.02	0.81 ± 0.05	0.13 ± 0.04	n.d.
Kaempferol	0.18 ± 0.03	1.17 ± 0.08	n.d.	n.d.
Tyrosol	n.d.	0.02 ± 0.00	n.d.	n.d.
Phloretic acid	n.d.	0.86 ± 0.05	n.d.	n.d.
Syringic acid	n.d.	0.91 ± 0.09	n.d.	n.d.
Sinapic acid	n.d.	0.24 ± 0.03	n.d.	n.d.
Naringenin	n.d.	1.81 ± 0.14	n.d.	n.d.
Chlorogenic acid	n.d.	116.98 ± 13.46	6.54 ± 0.98	n.d.
Resveratrol	6.49 ± 0.21	n.d.	n.d.	n.d.
3-4 dihydroxyphenylacetic acid	0.76 ± 0.08	n.d.	n.d.	n.d.

Data are presented as the average values of three replications ± standard deviation. S.Ch.: Sour cherry, Ch: Cherry, E.: Extract, D.E.: Dehydrated Extract. n.d.: not detected.

**Table 3:** Texture parameters of dough in control and enriched samples, as a function of proofing conditions and enrichment source (Ch., S.Ch.).

Proofing Conditions	Biological Leavening			Chemical Leavening		
Samples	Hardness (N)	Cohesiveness	Gumminess	Hardness (N)	Cohesiveness	Gumminess
C.	0.32 ± ${0.10\;}_{N.S}^{a}$	0.75 ± ${0.02\;}_{A}^{a}$	0.24 ± ${0.05\;}_{N.S}^{a.b}$	0.35 ± ${0.04\;}_{N.S}^{b}$	0.69 ± ${0.00\;}_{A}^{a}$	0.25 ± ${0.03\;}_{N.S}^{c.d}$
	Cherry					
E.	0.74 ± ${0.11\;}_{A}^{c}$	0.45 ± ${0.01\;}_{B}^{c}$	0.34 ± ${0.05\;}_{A}^{c}$	0.33 ± ${0.02\;}_{A}^{b}$	0.57 ± ${0.07\;}_{B}^{b}$	0.19 ± ${0.01\;}_{A}^{b}$
P.	0.33 ± ${0.03\;}_{B}^{a}$	0.71 ± ${0.02\;}_{C}^{a.b}$	0.23 ± ${0.02\;}_{N.S}^{a.b}$	0.45 ± ${0.02\;}_{B}^{c}$	0.59 ± ${0.07\;}_{C}^{b}$	0.26 ± ${0.02\;}_{N.S}^{d.e}$
E./P.	0.68 ± ${0.02\;}_{C}^{c}$	0.52 ± ${0.09\;}_{N.S}^{c}$	0.39 ± ${0.04\;}_{B}^{c}$	0.26 ± ${0.01\;}_{C}^{a}$	0.56 ± ${0.01\;}_{N.S}^{b}$	0.14 ± ${0.01\;}_{B}^{a}$
D.E.	0.42 ± ${0.04\;}_{D}^{a.b}$	0.65 ± ${0.03\;}_{N.S}^{b}$	0.26 ± ${0.04\;}_{N.S}^{b}$	0.32 ± ${0.01\;}_{D}^{b}$	0.62 ± ${0.10\;}_{N.S}^{a.b}$	0.21 ± ${0.05\;}_{N.S}^{b.c}$
D.E./P.	0.52 ± ${0.03\;}_{N.S}^{b}$	0.34 ± ${0.03\;}_{D}^{d}$	0.18 ± ${0.01\;}_{C}^{a.b}$	0.55 ± ${0.03\;}_{N.S}^{d}$	0.55 ± ${0.03\;}_{D}^{b}$	0.30 ± ${0.00\;}_{C}^{e}$
	Sour Cherry					
E.	0.63 ± ${0.01\;}_{A}^{d}$	0.64 ± ${0.01\;}_{B}^{c}$	0.37 ± ${0.03\;}_{A}^{c}$	0.36 ± ${0.02\;}_{A}^{a}$	0.58 ± ${0.03\;}_{B}^{b.c}$	0.21 ± ${0.01\;}_{A}^{b.c}$
P.	0.34 ± ${0.03\;}_{B}^{a}$	0.76 ± ${0.01\;}_{C}^{a}$	0.26 ± ${0.03\;}_{B}^{b}$	0.47 ± ${0.04\;}_{B}^{b}$	0.69 ± ${0.03\;}_{C}^{a}$	0.33 ± ${0.02\;}_{B}^{d}$
E./P.	0.65 ± ${0.02\;}_{C}^{d}$	0.63 ± ${0.02\;}_{N.S}^{c}$	0.43 ± ${0.03\;}_{C}^{d}$	0.33 ± ${0.01\;}_{C}^{a}$	0.58 ± ${0.04\;}_{N.S}^{b}$	0.19 ± ${0.02\;}_{C}^{a.b}$
D.E.	0.22 ± ${0.01\;}_{D}^{b}$	0.72 ± ${0.04\;}_{D}^{a.b}$	0.16 ± ${0.02\;}_{N.S}^{a}$	0.34 ± ${0.02\;}_{D}^{a}$	0.49 ± ${0.10\;}_{D}^{c}$	0.16 ± ${0.02\;}_{N.S}^{a}$
D.E./P.	0.41 ± ${0.03\;}_{N.S}^{c}$	0.68 ± ${0.06\;}_{E}^{b.c}$	0.21 ± ${0.01\;}_{N.S}^{a.b}$	0.38 ± ${0.03\;}_{N.S}^{a}$	0.49 ± ${0.04\;}_{E}^{b.c}$	0.20 ± ${0.03\;}_{N.S}^{b}$

Data are presented as the average values of three replications ± standard deviation. Lowercase letters indicate significant differences (*p* < 0.05) between samples; uppercase letters indicate significant differences between proofing conditions; N.S. indicate not significant differences between proofing conditions; C.: Control sample; E.: Extract; D.E.: Dehydrated Extract; P.: Pulverized Fruit; E./P.: Extract combined with pulverized fruit; D.E./P.: Dehydrated Extract combined with pulverized fruit.

**Table 4:** Texture parameters of bread crust in control and enriched samples, as a function of proofing conditions and enrichment source (Ch., S.Ch.).

Proofing Conditions	Biological Leavening			Chemical Leavening		
Samples	Hardness (N)	Cohesiveness	Gumminess	Hardness (N)	Cohesiveness	Gumminess
C.	10.3 ± ${0.1\;}_{A}^{a}$	0.70 ± ${0.00\;}_{A}^{a}$	7.2 ± ${0.1\;}_{A}^{b}$	15.2 ± ${0.8\;}_{A}^{a}$	0.61 ± ${0.03\;}_{A}^{a}$	9.2 ± ${0.1\;}_{A}^{a.b}$
	Cherry					
E.	42.6 ± ${3.9\;}_{B}^{d}$	0.30 ± ${0.02\;}_{N.S}^{a}$	12.6 ± ${0.8\;}_{B}^{d}$	28.9 ± ${2.5\;}_{B}^{b}$	0.33 ± ${0.04\;}_{N.S}^{b}$	9.5 ± ${0.5\;}_{B}^{b}$
P.	8.5 ± ${1.0\;}_{C}^{a}$	0.66 ± ${0.02\;}_{B}^{c}$	5.6 ± ${0.5\;}_{C}^{a}$	17.5 ± ${2.1\;}_{C}^{a}$	0.52 ± ${0.02\;}_{B}^{c}$	9.1 ± ${1.2\;}_{C}^{a.b}$
E./P.	29.3 ± ${0.9\;}_{N.S}^{c}$	0.30 ± ${0.00\;}_{N.S}^{c}$	8.8 ± ${0.4\;}_{N.S}^{c}$	29.8 ± ${6.3\;}_{N.S}^{b}$	0.30 ± ${0.03\;}_{N.S}^{c.d\;}$	8.6 ± ${2.4\;}_{N.S}^{a.b\;}$
D.E.	8.5 ± ${0.6\;}_{D}^{a}$	0.65 ± ${0.08\;}_{N.S}^{a}$	5.6 ± ${1.0\;}_{N.S}^{a}$	17.8 ± ${2.4\;}_{D}^{a}$	0.51 ± ${0.06\;}_{N.S}^{b}$	9.2 ± ${2.2\;}_{N.S}^{a.b}$
D.E./P.	20.0 ± ${2.0\;}_{E}^{b}$	0.45 ± ${0.09\;}_{N.S}^{b}$	8.9 ± ${1.3\;}_{D}^{c}$	25.8 ± ${0.4\;}_{E}^{b}$	0.26 ± ${0.01\;}_{N.S}^{d}$	6.6 ± ${0.1\;}_{D}^{a}$
	Sour Cherry					
E.	31.0 ± ${4.0\;}_{N.S}^{c}$	0.22 ± ${0.02\;}_{B}^{e}$	6.8 ± ${0.4\;}_{N.S}^{a}$	25.3 ± ${2.0\;}_{N.S}^{a}$	0.31 ± ${0.01\;}_{B}^{c}$	6.8 ± ${2.3\;}_{N.S}^{a}$
P.	12.3 ± ${1.6\;}_{N.S}^{a.b}$	0.69 ± ${0.02\;}_{C}^{a}$	8.5 ± ${0.9\;}_{N.S}^{a.b}$	15.4 ± ${1.5\;}_{N.S}^{a}$	0.56 ± ${0.02\;}_{C}^{a}$	8.6 ± ${0.9\;}_{N.S}^{a}$
E./P.	32.8 ± ${1.1\;}_{N.S}^{c}$	0.27 ± ${0.02\;}_{N.S}^{d}$	9.0 ± ${1.0\;}_{B}^{a.b}$	24.5 ± ${2.4\;}_{N.S}^{a}$	0.28 ± ${0.01\;}_{N.S}^{c}$	6.8 ± ${0.7\;}_{B}^{a}$
D.E.	15.2 ± ${0.8\;}_{B}^{b}$	0.61 ± ${0.01\;}_{N.S}^{b}$	9.2 ± ${0.3\;}_{C}^{b}$	11.1 ± ${2.3\;}_{B}^{a}$	0.59 ± ${0.06\;}_{N.S}^{a}$	6.4 ± ${0.7\;}_{C}^{a}$
D.E./P.	43.7 ± ${4.1\;}_{C}^{d}$	0.48 ± ${0.03\;}_{N.S}^{c}$	20.8 ± ${2.5\;}_{N.S}^{c}$	81.8 ± ${3.4\;}_{C}^{b}$	0.45 ± ${0.06\;}_{N.S}^{b}$	37.2 ± ${1.9\;}_{N.S}^{b}$

Data are presented as the average values of three replications ± standard deviation. Lowercase letters indicate significant differences (*p* < 0.05) between samples; uppercase letters indicate significant differences between proofing conditions; N.S. indicate not significant differences between proofing conditions; C.: Control sample; E.: Extract; D.E.: Dehydrated Extract; P.: Pulverized Fruit; E./P.: Extract combined with pulverized fruit; D.E./P.: Dehydrated Extract combined with pulverized fruit.

## Data Availability

The original contributions presented in the study are included in the article, further inquiries can be directed to the corresponding author.

## Appendix A

**Table A1:** Composition of prepared dough formulations.

Ingredients (%)	C.	E.	D.E.	P.	E./P.	D.E./P.	C.	E.	D.E.	P.	E./P.	D.E./P.
	Biological Leavening						Chemical Leavening					
Wheat Flour	54.2	54.2	52.3	51.3	52.3	50.3	53.5	53.5	51.2	51.2	51.2	49.2
Sugar	7	7	7	7	7	7	7	7	7	7	7	7
Salt	1	1	1	1	1	1	1	1	1	1	1	1
Olive Oil	7	7	7	7	7	7	7	7	7	7	7	7
Dry Yeast	0.7	0.7	0.7	0.7	0.7	0.7						
Baking Powder							1.8	1.8	1.8	1.8	1.8	1.8
Water	30.1			30		29	29.7			29		29
Extract		30.1	30		29			29.7	30		29	
Dehydrated Extract	-		2			2	-		2			2
Pulverized Fruit				3	3	3				3	3	3

C.: Control sample; E.: Extract; D.E.: Dehydrated Extract; P.: Pulverized Fruit; E./P.: Extract combined with pulverized fruit; D.E./P.: Dehydrated Extract combined with pulverized fruit.

**Table A2:** Color parameters (L*, a*, and b*), water activity and moisture content, and % swelling of dough, enriched with 5 different forms using 2 methods of swelling (biological (*S. cerevisiae*) and chemical (sodium acid pyrophosphate—sodium bicarbonate).

		Cherry Dough						Sour Cherry Dough					
		L*	a*	b*	aw	Moisture % d.w.	% Swelling	L*	a*	b*	aw	Moisture % d.w.	% Swelling
C.	Chemical Leavening	72.3 ± 0.3 ^f^	3.74 ± 0.07 ^a^	29.66 ± 0.09 ^f^	0.977 ± 0.000 ^c^	46.9% ± 0.8 ^b^	11.00 ± 5.07 ^a,b^	72.2 ± 0.0 ^f^	2.79 ± 0.01 ^a^	25.45 ± 0.22 ^f^	0.965 ± 0.002 ^d^	50.7% ± 0.1 ^d^	8.75 ± 0.63 ^b,c^
E.		44.8 ± 0.0 ^c^	10.08 ± 0.02 ^e^	12.58 ± 0.02 ^c^	0.883 ± 0.004 ^a^	46.4% ± 0.7 ^b^	18.77 ± 5.37 ^c^	52.7 ± 0.0 ^c^	9.65 ± 0.02 ^d^	11.56 ± 0.02 ^c^	0.944 ± 0.001 ^c^	47.4% ± 0.3 ^b^	13.15 ± 0.81 ^c^
D.E.		54.4 ± 0.0 ^d^	7.01 ± 0.02 ^b^	14.73 ± 0.02 ^d^	0.976 ± 0.003 ^b^	50.4% ± 0.5 ^c^	9.11 ± 0.89 ^a^	59.6 ± 0.0 ^d^	6.75 ± 0.01 ^c^	13.56 ± 0.06 ^d^	0.989 ± 0.005 ^a^	50.0% ± 0.2 ^b.c^	6.37 ± 0.42 ^b^
P.		48.6 ± 0.0 ^a^	6.08 ± 0.01 ^f^	13.65 ± 0.01 ^a^	0.905 ± 0.004 ^b^	50.6% ± 0.3 ^a^	9.58 ± 0.47 ^a^	53.2 ± 0.0 ^a^	8.83 ± 0.02 ^f^	13.09 ± 0.04 ^b^	0.909 ± 0.001 ^b^	53.8% ± 0.2 ^a^	11.43 ± 2.89 ^b,c^
E./P.		37.5 ± 0.0 ^e^	10.67 ± 0.02 ^c^	9.20 ± 0.03 ^e^	0.903 ± 0.005 ^c^	44.5% ± 0.7 ^c^	16.87 ± 2.96 ^b,c^	40.5 ± 0.0 ^e^	18.86 ± 0.06 ^b^	8.26 ± 0.03 ^e^	0.925 ± 0.002 ^e^	48.3% ± 2.2 ^e^	21.23 ± 1.58 ^d^
D.E/P.		41.8 ± 0.0 ^b^	8.66 ± 0.00 ^d^	10.40 ± 0.02 ^b^	0.980 ± 0.000 ^c^	51.3% ± 0.4 ^c^	7.06 ± 3.93 ^a^	42.8 ± 0.3 ^b^	18.19 ± 0.11 ^e^	6.94 ± 0.09 ^a^	0.926 ± 0.002 ^b^	54.9% ± 0.2 ^c^	0.77 ± 0.00 ^a^
C.	Biological Leavening	72.3 ± 0.3 ^f^	3.74 ± 0.07 ^a^	29.66 ± 0.09 ^f^	0.977 ± 0.002 ^d^	50.5% ± 0.8 ^c^	122.4 ± 3.8 ^e^	72.0 ± 0.0 ^f^	2.94 ± 0.02 ^a^	26.00 ± 0.14 ^f^	0.997 ± 0.001 ^e^	53.0% ± 0.6 ^b^	120.83 ± 5.47 ^b^
E.		44.8 ± 0.0 ^c^	10.08 ± 0.02 ^e^	12.58 ± 0.02 ^c^	0.919 ± 0.001 ^a^	47.9% ± 0.8 ^b^	1.6 ± 0.6 ^a^	49.5 ± 0.1 ^c^	16.22 ± 0.03 ^c^	10.67 ± 0.10 ^d^	0.818 ± 0.003 ^b^	49.9% ± 0.2 ^b^	4.13 ± 1.15 ^a^
D.E.		54.4 ± 0.0 ^d^	7.01 ± 0.02 ^b^	14.73 ± 0.02 ^d^	0.973 ± 0.000 ^c^	50.3% ± 0.7 ^c^	87.3 ± 10.2 ^c^	61.4 ± 0.0 ^d^	10.18 ± 0.01 ^d^	14.33 ± 0.05 ^c^	0.967 ± 0.006 ^c^	51.5% ± 0.9 ^b^	94.12 ± 7.59 ^b^
P.		48.6 ± 0.0 ^a^	6.08 ± 0.01 ^f^	13.65 ± 0.01 ^a^	0.947 ± 0.011 ^b^	50.1% ± 0.3 ^a^	99.5 ± 5.1 ^d^	50.3 ± 0.1 ^a^	18.01 ± 0.02 ^f^	9.61 ± 004 ^a^	0.898 ± 0.008 ^a^	51.3% ± 1.4 ^a^	8.75 ± 0.63 ^b,c^
E./P.		37.5 ± 0.0 ^e^	10.67 ± 0.02 ^c^	9.20 ± 0.03 ^e^	0.916 ± 0.004 ^c.d^	46.2% ± 1.00 ^c^	1.2 ± 0.8 ^a^	39.8 ± 0.1 ^e^	24.89 ± 0.04 ^b^	8.79 ± 0.02 ^e^	0.800 ± 0.004 ^d^	47.7% ± 0.4 ^a^	13.15 ± 0.81 ^c^
D.E./P.		41.8 ± 0.0 ^b^	8.66 ± 0.00 ^d^	10.40 ± 0.02 ^b^	0.907 ± 0.001 ^a^	52.5% ± 0.44 ^d^	53.5± 0.4 ^b^	45.5 ± 0.1 ^b^	22.35 ± 0.04 ^e^	9.18 ± 0.06 ^b^	0.895 ± 0.001 ^c^	53.6% ± 0.3 ^b^	6.37 ± 0.42 ^b^

Data are presented as the average values of three replications ± standard deviation. Lowercase letters indicate significant differences (*p* < 0.05) at a 95% confidence level. C.: Control sample; E.: Extract; D.E.: Dehydrated Extract; P.: Pulverized Fruit; E./P.: Extract combined with pulverized fruit; D.E./P.: Dehydrated Extract combined with pulverized fruit.

**Table A3:** Phase angle δ (tanδ), Complex viscosity (η*. Pa·s), Storage Modulus (G′. Pa), and Loss Modulus (G″. Pa) values at a frequency of 1 Hz in control and enriched samples as a function of proofing conditions and enrichment source (Ch. S.Ch.).

Proofing Conditions	Biological Leavening				Chemical Leavening			
Samples	Tan δ	Complex Viscosity (η*, Pa·s)	Storage Modulus, G′ (Pa)	Loss Modulus, G″ (Pa)	Tan δ	Complex Viscosity (η*, Pa·s)	Storage Modulus, G′ (Pa)	Loss Modulus, G″ (Pa)
C.	0.493 ± 0.06 ^b,b^ *	3081 ± 58 ^b,b^ *	17,438 ± 81 ^b,c^ *	8598 ± 83 ^b,c^ *	0.478 ±0.01 ^b,a^ *	5271 ± 121 ^c,d^ *	29,765 ± 18 ^b,d^ *	14,213 ± 93 ^c.,c^ *
Ch.E.	0.443 ± 0.06 ^a^	3230 ± 15 ^b^	20,802 ± 181 ^b^	9199 ± 91 ^b^	0.443 ± 0.06 ^a^	4137 ± 68 ^b^	27,161 ± 116 ^b^	10,884 ± 89 ^b^
Ch.P.	0.493 ± 0.021 ^b^	2112 ± 10 ^a^	11,962 ± 64 ^a^	5868 ± 71 ^a^	0.477 ± 0.01 ^b^	2377 ± 38 ^a,b^	12,147 ± 121 ^a^	6029 ± 34 ^a^
Ch.E./P.	0.433 ± 0.06 ^a^	4740 ± 67 ^c^	27′451 ± 121 ^d^	11,876 ± 33 ^c^	0.447 ± 0.02 ^a^	3748 ± 97 ^b^	33,194 ± 174 ^b^	14,964 ± 117 ^c^
Ch.D.E.	0.533 ± 0.06 ^c^	1914 ± 86 ^a^	10,663 ± 61 ^a^	5666 ± 36 ^a^	0.52 ± 0.02 ^c^	1620 ± 70 ^a^	13,970 ± 94 ^a^	7205 ± 46 ^a^
Ch.D.E./P.	0.513 ± 0.06 ^c^	4192 ± 92 ^c^	23,553 ± 142 ^c.d^	12,033 ± 85 ^c^	0.53 ± 0.02 ^c^	2388 ± 36 ^a,b^	30,661 ± 143 ^b^	16,833 ± 67 ^c^
S.Ch.E.	0.443 ± 0.04 ^a^*	4421 ± 53 ^c^*^8^	23,875 ± 56 ^d^*	10,548 ± 62 ^d^*	0.46 ± 0.04 ^a^*	2792 ± 29 ^b^*	19,240 ± 122 ^b^*^,c^*	8938 ± 45 ^b^*
S.Ch.P.	0.46 ± 0.03 ^b^*	2009 ± 63 ^a^*	13,583 ± 102 ^b^*	6458 ± 18 ^b^*	0.50 ± 0.02 ^a^*^,b^*	2636 ± 55 ^b^*	14,909 ± 81 ^b^*	7366 ± 34 ^b^*
S.Ch.E./P.	0.453 ± 0.03 ^a^*^,b^*	5772 ± 31 ^d^*	21,595 ± 138 ^d^*	9640 ± 84 ^c^*^,d^*	0.51 ± 0.01 ^b^*	4304 ± 10 ^c^*	24,217 ± 123 ^c^*	12,283 ± 130 ^c^*
S.Ch.D.E.	0.517 ± 0.00 ^c^*	2491 ± 19 ^a^*	9068 ± 53 ^a^*	4715 ± 52 ^a^*	0.47 ± 0.01 ^a^*	1828 ± 47 ^a^*	9420 ± 39 ^a^*	4486 ± 66 ^a^*
S.Ch.D.E./P.	0.52 ± 0.00 ^c^*	6063 ± 99 ^d^*	13,317 ± 74 ^b^*	7037 ± 25 ^b^*	0.49 ± 0.01 ^a^*^,b^*	2498 ± 23 ^b^*	14,139 ± 103 ^a^*^,b^*	6969 ± 43 ^b^*

Data are presented as the average values of three replications ± standard deviation. Lowercase letters indicate significant differences (*p* < 0.05) between samples enriched with Cherry formulations; lowercase letters with asterisk (*) indicate significant differences between samples enriched with Sour Cherry formulations; C.: Control sample; E.: Extract; D.E.: Dehydrated Extract; P.: Pulverized Fruit; E./P.: Extract combined with pulverized fruit; D.E./P.: Dehydrated Extract combined with pulverized fruit.
